# Investigating amygdala habituation in major depressive disorder: an fMRI study in UK Biobank

**DOI:** 10.1192/j.eurpsy.2025.465

**Published:** 2025-08-26

**Authors:** L. Fortaner-Uyà, C. Verga, S. Cademartiri, E. Tassi, P. Brambilla, E. Maggioni, C. Fabbri, F. Benedetti, B. Vai

**Affiliations:** 1 University Vita-Salute San Raffaele; 2 Division of Neuroscience, IRCCS San Raffaele Hospital, Psychiatry and Clinical Psychobiology Unit; 3 Department of Neurosciences and Mental Health, Fondazione IRCCS Ca’ Granda Ospedale Maggiore Policlinico; 4 Department of Electronics - Information and Bioengineering, Politecnico di Milano, Milan; 5 Department of Biomedical and Neuromotor Sciences, University of Bologna, Bologna, Italy

## Abstract

**Introduction:**

Major depressive disorder (MDD) is a severe psychiatric condition with a high risk of suicide. Research on MDD and suicidality has identified structural and functional abnormalities in the cortico-limbic network as candidate biomarkers, but little is known about the temporal dynamics of these brain regions. Recently, abnormal amygdala habituation to emotional stimuli has been highlighted as a reliable fMRI phenotype linked to emotional dysregulation and increased suicide risk.

**Objectives:**

Our study aimed to assess amygdala habituation to emotional stimuli in MDD and explore differences between suicide attempters (SA) and non-attempters (nSA). Additionally, we examined the relationship between amygdala habituation and depressive symptoms.

**Methods:**

414 MDD patients (239 SA, 175 nSA) selected from the UK Biobank underwent fMRI during a block-designed emotion processing task, including faces and shapes conditions. We obtained bilateral amygdala activation for each block using FSL. Habituation was quantified using two methods: the regression approach (REG) and First minus Last block (FmL). One sample T-tests were used to investigate whether habituation rates significantly differed from zero. Group differences were analysed using Mann-Whitney U-tests. Generalized linear models (GLM) were applied to examine relationships between habituation and depression severity, controlling for age, sex, group (SA vs. nSA), and handedness.

**Results:**

In both MDD and SA groups, no significant habituation was observed for either emotional or non-emotional stimuli (p*_FDR_* >.05). However, the nSA group showed significantly positive habituation rates for left amygdala in both conditions and for right amygdala in faces condition using REG (p*_FDR_* <.05), suggesting a possible sensitization process. Moreover, nSA showed significantly higher habituation rates than SA in all conditions with REG (p*_FDR_* <.01). GLM analyses revealed no significant associations with depression severity.

**Conclusions:**

Our results suggest that MDD is characterized by a lack of amygdala habituation to emotional stimuli, potentially offering new insights into its pathophysiology. This biomarker may help in developing novel therapeutic strategies targeting the amygdala and its regulation within the cortico-limbic system.

**Fundings:**

The current study was supported by the Italian Ministry of Health, GR-2019-12370616.

**Disclosure of Interest:**

None Declared

